# Sodium–Glucose Co-Transporter 2 Inhibition With Empagliflozin Improves Cardiac Function After Cardiac Arrest in Rats by Enhancing Mitochondrial Energy Metabolism

**DOI:** 10.3389/fphar.2021.758080

**Published:** 2021-10-12

**Authors:** Yunke Tan, Kai Yu, Lian Liang, Yuanshan Liu, Fengqing Song, Qiulin Ge, Xiangshao Fang, Tao Yu, Zitong Huang, Longyuan Jiang, Peng Wang

**Affiliations:** ^1^ Department of Emergency Medicine, Sun Yat-sen Memorial Hospital, Sun Yat-sen University, Guangzhou, China; ^2^ Institute of Cardiopulmonary Cerebral Resuscitation, Sun Yat-sen University, Guangzhou, China

**Keywords:** cardiac arrest, cardiopulmonary resuscitation, empagliflozin, mitochondria, ketone body

## Abstract

Empagliflozin is a newly developed antidiabetic drug to reduce hyperglycaemia by highly selective inhibition of sodium–glucose co-transporter 2. Hyperglycaemia is commonly seen in patients after cardiac arrest (CA) and is associated with worse outcomes. In this study, we examined the effects of empagliflozin on cardiac function in rats with myocardial dysfunction after CA. Non-diabetic male Sprague–Dawley rats underwent ventricular fibrillation to induce CA, or sham surgery. Rats received 10 mg/kg of empagliflozin or vehicle at 10 min after return of spontaneous circulation by intraperitoneal injection. Cardiac function was assessed by echocardiography, histological analysis, molecular markers of myocardial injury, oxidative stress, mitochondrial ultrastructural integrity and metabolism. We found that empagliflozin did not influence heart rate and blood pressure, but left ventricular function and survival time were significantly higher in the empagliflozin treated group compared to the group treated with vehicle. Empagliflozin also reduced myocardial fibrosis, serum cardiac troponin I levels and myocardial oxidative stress after CA. Moreover, empagliflozin maintained the structural integrity of myocardial mitochondria and increased mitochondrial activity after CA. In addition, empagliflozin increased circulating and myocardial ketone levels as well as heart *β*-hydroxy butyrate dehydrogenase 1 protein expression. Together, these metabolic changes were associated with an increase in cardiac energy metabolism. Therefore, empagliflozin favorably affected cardiac function in non-diabetic rats with acute myocardial dysfunction after CA, associated with reducing glucose levels and increasing ketone body oxidized metabolism. Our data suggest that empagliflozin might benefit patients with myocardial dysfunction after CA.

## Introduction

Cardiac arrest (CA) is an important public health challenge, and it seems to account for approximately 50% of all cardiovascular deaths (Fishman et al., 2010). Cardiopulmonary resuscitation (CPR) is an effective treatment to return of spontaneous circulation (ROSC) for CA victims. Patients who initially achieve ROSC after CA have high morbidity and mortality rates due to post-CA syndrome, including brain injury, myocardial dysfunction, and systemic inflammatory responses (Nolan et al., 2008; Girotra et al., 2012). Post-CA myocardial dysfunction, including arterial hypotension, ventricular arrhythmias, and recurrent CA has been recognized as the leading cause of early death after ROSC (Nolan et al., 2008; Patil et al., 2015). Although therapeutic hypothermia has proved effective in clinical studies, pharmacological treatment options presently for post-CA myocardial dysfunction are limited (Hypothermia after Cardiac Arrest Study, 2002).

Post-CA myocardial dysfunction is associated with global myocardial ischemia/reperfusion. Main contributors to ischemia/reperfusion injury include ATP depletion and generation of reactive oxygen species (ROS) (Patil et al., 2015; Yang et al., 2015). Recent studies aimed at protecting mitochondria from reperfusion injury during ROSC indicated that preservation of mitochondrial bioenergetic function in the heart helps restoration of cardiac activity after CA (Ayoub et al., 2008; Gazmuri and Radhakrishnan, 2012). In addition, glucose homeostasis is important in the pathophysiology of critical illness and can be used as a determinant of outcome following resuscitation from CA. Hyperglycaemia is commonly seen in patients after CA and is associated with worse outcomes (Beiser et al., 2009; Nurmi et al., 2012; Ettleson et al., 2014). Several clinical studies have suggested that treatment of hyperglycemia may improve morbidity and mortality in critically-ill patients (Van den Berghe et al., 2006; Vogt et al., 2014). However, some other studies have shown that tight glucose control through intensive insulin therapy may not necessarily improve outcomes in CA patients (Oksanen et al., 2007; Losert et al., 2008).

Sodium glucose cotransporter 2 (SGLT2) is a channel protein primarily found in the proximal convoluted tubule of the kidney and regulates renal tubular glucose reabsorption using the gradient of the sodium ions concentration between inside and outside of the cells. Therefore, SGLT2 inhibitor can reduce glucose reabsorption and lower the blood glucose (de Leeuw and de Boer, 2016). Empagliflozin, a highly selective SGLT2 inhibitor, is a newly developed oral antidiabetic drug to reduce hyperglycaemia in an insulin-independent manner. Beyond glucose control, recent studies found that SGLT2 inhibitor has a potential protective effect against cardiovascular events. Empagliflozin has demonstrated to reduce cardiovascular mortality by 38% and heart failure hospitalizations by 35% in patients with type 2 diabetes mellitus in the EMPA-REG OUTCOMES trial (Fitchett et al., 2016). Furthermore, emerging evidence demonstrated the effectiveness of empagliflozin in non-diabetic heart failure (Santos-Gallego et al., 2019; Yurista et al., 2019; Packer et al., 2020). The actual mechanisms responsible for these beneficial effects in heart failure are not completely clear. Thus far, a number of mechanisms have been proposed to explain the cardioprotective effects of SGLT2 inhibitors, including beneficial effects on cardiac energy metabolism, reducing inflammation, blood pressure reduction, improving kidney function, decreasing oxidative stress, and so on (Lopaschuk and Verma, 2020). However, the effect of empagliflozin on post-CA myocardial dysfunction in non-diabetic hearts and involved mechanisms remain unknown.

In the present study, we therefore investigated the effects of empagliflozin on acute myocardial injury after CA and dissected the involved molecular mechanism in non-diabetic hearts. We hypothesized that empagliflozin could alleviate myocardial injury after CA by maintaining glucose homeostasis and protecting mitochondrial function. Here, we examined the effects of empagliflozin on myocardial injury in a non-diabetic rat model of ventricular fibrillation CA and CPR.

## Materials and Methods

### Animals and Drugs

Male Sprague-Dawley rats (330–355 g) were obtained from Experimental Animal Center of Sun Yat-sen University (Guangzhou, China). Animals were maintained on laboratory chow in a specific pathogen-free room at a constant temperature (20–22°C) with 12 h of light and 12 h of dark exposure. All animal experiments were performed in accordance with the ARRIVE guideline (Kilkenny et al., 2010). All animal studies were approved by the Institutional Animal Care and Use Committee of Sun Yat-sen University (SYSU-IACUC-2019-B73).

Empagliflozin was purchased from MedChemExpress lnc (Monmouth Junction, United States). Empagliflozin was dissolved in 10% dimethyl sulfoxide, 20% kolliphor (Sigma Aldrich, United States) and 80% phosphate buffer saline as a stock solution (10 mg/ml).

### CA Model and Experimental Protocol

The animals were surgically prepared as previously described (Wang et al., 2018). CA was induced by ventricular fibrillation (VF) through a transoesophageal electrode. The electrode was positioned to ensure constant ventricular capture and was given a 30 V voltage and 30 Hz current. The current flow continued for 3 min to prevent a spontaneous reversal of VF. After 6 min of untreated VF, CPR was started with precordial compression (PC) at a rate of 250/minutes and mechanical ventilation with 100% O_2_. After 2 min of PC, epinephrine (0.01 mg/kg) was administrated through the femoral artery and a 2 J biphasic waveform shock was performed after 4 min of CPR. ROSC was defined as the return of supraventricular rhythm with a mean artery pressure (MAP) ≥ 60 mmHg for a minimum of 5 min. If VF persisted, another 2 J shock was given after another 1 min of PC. Resuscitation was declared a failure when there was no ROSC after 6 min of CPR. After ROSC, mechanical ventilation was continued with 100% O_2_ for 30 min, 50% O_2_ for 20 min, and 30% O_2_ for 10 min.

**FIGURE 1:**
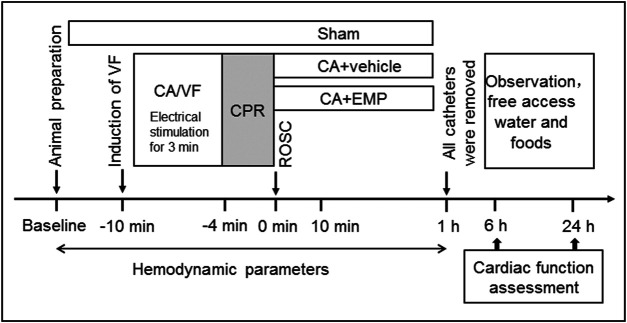
Experimental procedures in VF-induced cardiac arrest model. *VF* ventricular fibrillation, *CA* cardiac arrest, *CPR* cardiopulmonary resuscitation, *ROSC* return of spontaneous circulation, *EMP* empagliflozin.

The animals were randomized into three groups: CA + empagliflozin (EMP) group (*n* = 18), CA + vehicle group (*n* = 22) and Sham group (*n* = 6). The animals in Sham group underwent the same operation with the other groups but without inducing CA. The rats in the CA + EMP group received 10 mg/kg of empagliflozin at 10 min after ROSC by intraperitoneal injection. Meanwhile, the rats in Vehicle group received placebo (10% dimethyl sulfoxide, 20% kolliphor and 80% phosphate buffer saline) for control. An illustration of the protocol used is provided (Figure 1).

### Histopathologic Analysis

The heart tissues at 24 h after ROSC were embedded in paraffin and sectioned at 5 μm thickness. Tissue slices of the hearts were stained with Masson’s trichrome (Servicebio, Guangzhou, China) for histological evaluation according to the manufacturer’s instructions. Ten fields of vision (magnification of ×400) were randomly sampled from each heart tissue sample under an optical microscope. The fibrotic tissue area stained blue (%) by Masson staining was analyzed using counting software (Image J, National Institute of Health, United States). The Masson staining was evaluated by two investigators unaware of group identities.

### Serum Cardiac Troponin I Levels

Blood samples at 24 h after ROSC were collected from the abdominal aorta in rats and incubated at room temperature for 30 min to allow the blood to clot. The serum was separated by centrifugation at 1,500 *g* for 15 min and stored at −80°C until use. Serum cTnI concentrations were determined using a rat cTnI enzyme-linked immunosorbent assay kit (Cusabio, China) according to the manufacturer’s instructions.

### Echocardiography

Rats were anesthetized with 2–3% isoflurane, and *in vivo* cardiac function at 24 h after ROSC was assessed by transthoracic echocardiography. The M-mode and two-dimensional echocardiography was performed using a high-resolution imaging system equipped with a 30-MHz transducer (Vevo 3,100, VisualSonics, Toronto, Canada), as previously described (Yin et al., 2011). The left ventricular (LV) function was assessed by ejection fraction (EF) and cardiac output (CO). All measurements were conducted by a single investigator who was blinded to the experimental groups.

### Oxidative Stress Analysis

The oxidative stress in the heart tissue at 24 h after CA was evaluated by immunohistochemical staining of 4-hydroxynonenal (4-HNE) to assess lipid peroxidation and 8-hydroxy-2′-deoxyguanosine (8-OHdG) to detect the extent of nucleic acid oxidation. Heart tissues were sectioned and embedded in paraffin. For immunohistochemistry, the fixed sections were immunostained overnight at 4°C using a primary monoclonal antibody against 4-HNE and 8-OHdG (Abcam, Cambridge, United Kingdom), followed by corresponding secondary antibody for 2.5 h at room temperature. Ten fields of vision (magnification of ×400) were randomly sampled from each heart tissue sample under an optical microscope. 4-HNE and 8-OHdG relative intensity area (%) were detected by automated counting software (Image J, National Institute of Health, United States). The ROS levels in the heart at 6 h after CA were measured using a tissue ROS assay kit (Genmed Scientifics, Wilmington, DE, United States) according to the manufacturer’s instructions.

### Western Blot Analysis

For immunoblotting at 24 h after ROSC, *β*-hydroxy butyrate dehydrogenase 1 (BDH1) and dynamin-related protein 1 (Drp1) (diluted 1:1,000; Abcam, Cambridge, United Kingdom) and GAPDH (diluted 1:2000; Cell Signaling Technologies, United States) antibodies were used. GAPDH was used to normalize protein loading. The densities of protein blots were quantified by using Image J software (National Institutes of Health, Bethesda, United States) and normalized to control.

### Transmission Electron Microscopy and Analysis

Heart samples at 24 h after ROSC were collected and fixed in 2.5% glutaraldehyde in 0.1 mol/L phosphate buffer (pH 7.4) overnight at 4°C. The heart tissues were cut into 500-μm thick transverse slices and were post-fixed in 1% osmium tetroxide for 1 h. Tissue slices were then dehydrated in ascending series of ethanol and embedded in epon. After dehydration, the specimens were conventionally processed and examined under a transmission electron microscope (Tecnai G2, FEI, Hillsboro, United States) through 13,500× objectives. Area of individual mitochondria was quantified by using Image J software (National Institutes of Health, Bethesda, United States).

### Mitochondrial Respiration

Heart mitochondria at 6 and 24 h after ROSC were isolated using a Qproteome mitochondria isolation kit (Qiagen, Germany) according to the manufacturer’s instructions. Mitochondrial respiration was measured using a Clark oxygen electrode (Oxygraph, Hansatech Instruments, United Kingdom), as previously described (Wang et al., 2016). Freshly isolated mitochondria were incubated with glutamate-malate (2 mM) and succinate (4 mM) solution at 37°C. State III mitochondrial respiration was induced by the addition of adenosine 5′-diphosphate (ADP, 0.5 mmol/L). On ADP depletion, state IV respiration was measured. The mitochondrial respiratory control ratio (RCR) was calculated as the ratio of the respiratory rate in state III to that in state IV.

### Mitochondrial Complex Ⅰ Activity

The activity of mitochondrial complex Ⅰ at 6 and 24 h after ROSC was measured in heart homogenates using the complex I enzyme activity assay kit (Colorimetric) (Abcam, Cambridge, United Kingdom) according to the manufacturer’s instructions.

### Measurement of Metabolites Levels

Metabolites levels in heart were measured at 6 and 24 h after ROSC. ATP levels were measured in heart homogenates with an ATP bioluminescent assay kit (Beyotime, China) according to the manufacturer’s instructions. β-hydroxybutyrate (BHB) levels in heart and serum were detected by a colorimetric assay kit (Biovision, Milpitas, CA, United States) according to the manufacturer’s instructions. Nicotinamide adenine dinucleotide (NAD^+^)/reduced form of NAD^+^ (NADH) levels in heart homogenates were detected by a NAD^+^/NADH quantification colorimetric kit (Biovision, Milpitas, CA, United States) according to the manufacturer’s instructions. Glucose and lactate concentrations in the serum were measured by a biochemistry analyzer (YSI, Yellow Springs, OH, United States).

### Statistical Analysis

All data were presented as mean ± standard deviation (SD), and analyzed using GraphPad Prism 8 (GraphPad, San Diego, CA, United States). The unpaired t test was used to compare the same parameters between two groups. Normally distributed data were analyzed by one-way analysis of variance (ANOVA) followed by Tukey’s post hoc test. Lipid droplets number was analyzed by the Kruskal-Wallis test with Dunn’s post hoc analyses. The survival rates between the groups were compared using Kaplan-Meier survival analysis. A 2-sided *p* < 0.05 was considered significant.

## Results

### Animal Physiologic Data and Resuscitation Characteristics

A total of 52 rats were prepared for this study. Of these rats, six rats received sham operation (The Sham group), and 46 other rats underwent CPR but six rats failed to achieve ROSC. In the CA + vehicle group, 22 rats achieved ROSC but 10 rats failed to survive within 24 h after ROSC. In the CA + EMP group, 18 rats achieved ROSC but six rats failed to survive within 24 h after ROSC. The rats that failed to achieve ROSC were excluded from the group. There were no statistically significant differences in baseline physiologies, hemodynamic, cardiac function, and blood analytical measurements among the three groups (Supplementary Table S1). For rats that achieved ROSC, the time of CPR was comparative between the CA + vehicle and CA + EMP group (Supplementary Table S1).

### Empagliflozin Improved Cardiac Function After CA

**FIGURE 2:**
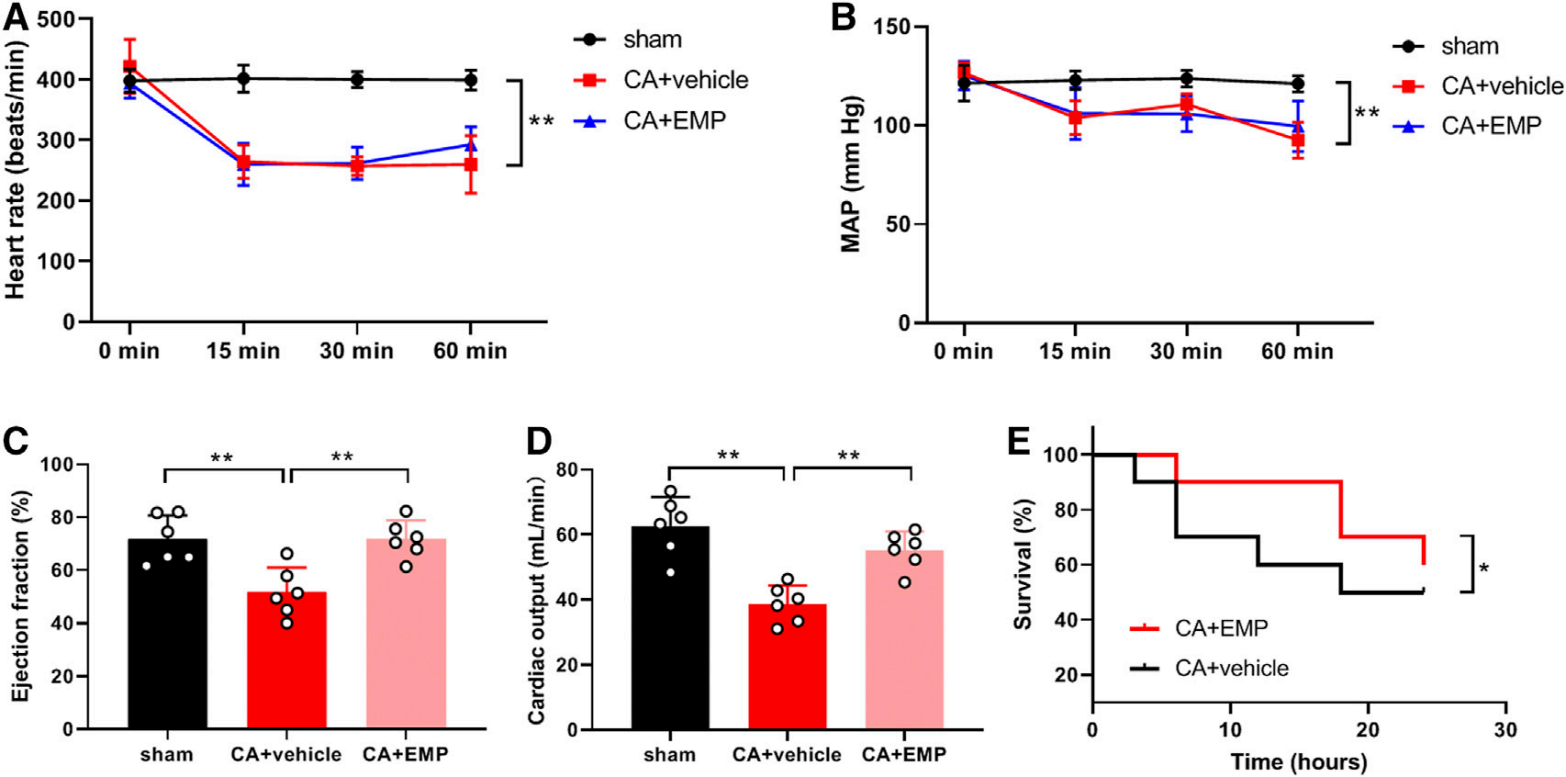
Empagliflozin (EMP) improved cardiac function after cardiac arrest in rats. **(A)** Effect of EMP on heart rate within 1 h after return of spontaneous circulation (ROSC) (*n* = 7–10). **(B)** Effect of EMP on mean artery pressure (MAP) within 1 h after ROSC (*n* = 7–10). **(C)** EMP increased left ventricular ejection fraction at 24 h after ROSC (*n* = 6). **(D)** EMP increased cardiac output at 24 h after ROSC (*n* = 6). **(E)** EMP improved survival of the rats after cardiac arrest within 24 h (*n* = 10). Data are presented as mean ± SD, *p < 0.05, **p < 0.01.

To explore the effect of empagliflozin on hemodynamic in rats, heart rate and blood pressure were detected at 15, 30 and 60 min after CA. Our results showed that both heart rate (Figure 2A) and blood pressure (Figure 2B) were significantly decreased within 1 h after ROSC (*p* < 0.01). Empagliflozin treatment had no effects on heart rate (Figure 2A) and blood pressure (Figure 2B) within 1 h after CA. In addition, the cardiac function was measured by Doppler echocardiography at 24 h after CA. We found that LVEF (Figure 2C) and CO (Figure 2D) were significantly decreased after CA (*p* < 0.01). However, empagliflozin treatment significantly increased both LVEF and CO (Figures 2C,D) (LVEF: 71.7 ± 7.1% versus 51.7 ± 9.3%, *p* < 0.01; CO: 55.2 ± 5.8% versus 38.7 ± 5.7%, *p* < 0.01). Moreover, Kaplan–Meier survival analysis showed that empagliflozin treatment significantly increased the survival time of the animals after CA within 24 h (*p* < 0.05) (Figure 2E).

### Empagliflozin Decreased Myocardial Injury After CA

**FIGURE 3:**
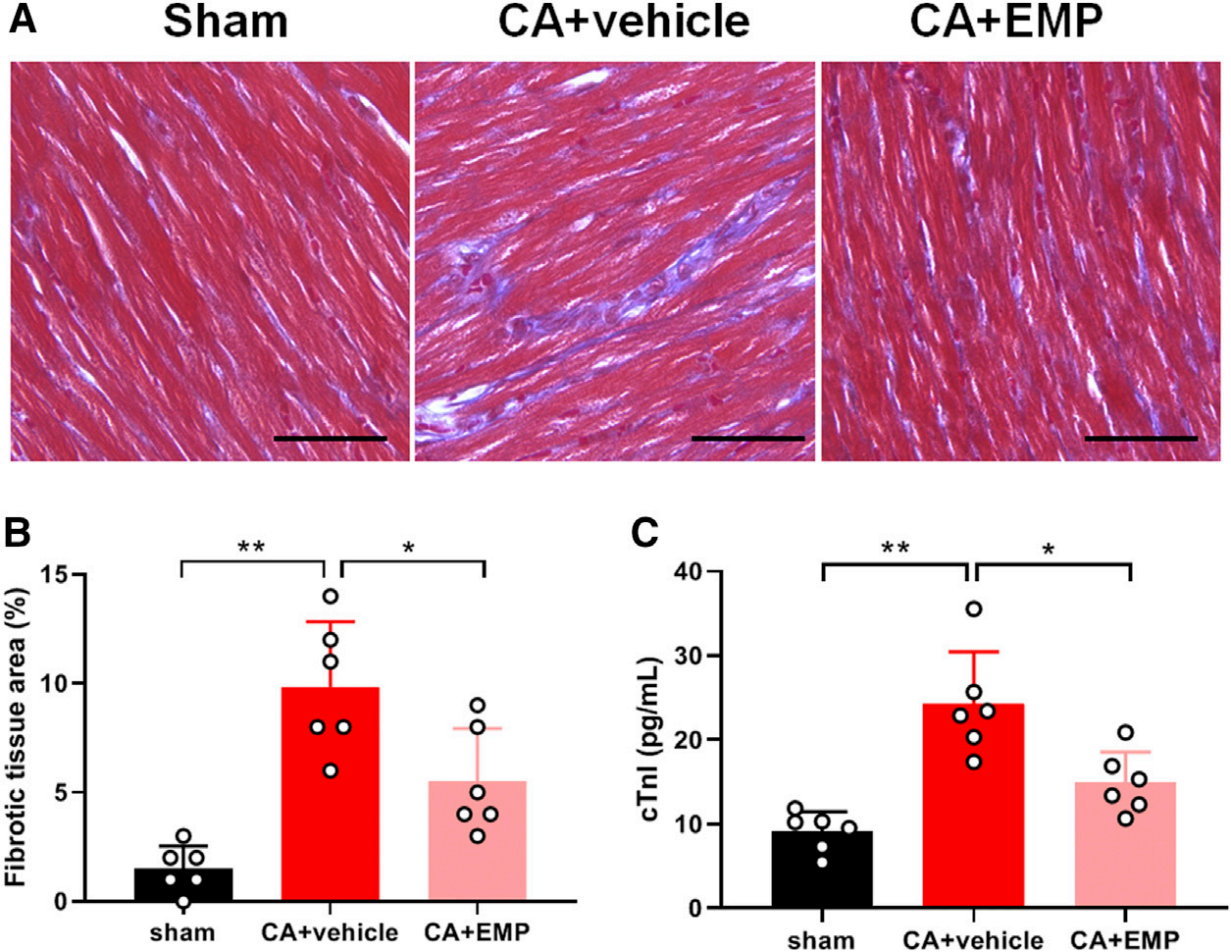
Empagliflozin (EMP) decreased myocardial injury after cardiac arrest in rats. **(A)** Representative photographs of Masson staining of heart sections obtained 24 h after return of spontaneous circulation (ROSC). Masson staining showed that EMP decreased myocardial fibrosis (stained blue) after ROSC. Scale bar = 50 μm. **(B)** Quantification of myocardial fibrotic area from Masson stained section. **(C)** EMP decreased serum cardiac troponin I (cTnI) level at 24 h after ROSC. Data are presented as mean ± SD, *n* = 6, *p < 0.05, **p < 0.01.

Histological analysis with Masson staining were performed to detect the myocardial injury 24 h after CA. Compared with the sham group, the myocardial fibrosis significantly increased in the CA + vehicle group (Figure 3A). However, the myocardial fibrosis was significantly lower in the CA + EMP group than that in the CA + vehicle group (6 ± 2% versus 10 ± 3%, *p* < 0.05) (Figure 3B). In addition, serum cTnI levels were detected in three groups 24 h after CA. The serum cTnI concentration increased significantly after ROSC (*p* < 0.01). However, the serum cTnI concentration in the CA + EMP group was significantly decreased compared with the CA + vehicle group (15 ± 4 pg/ml versus 20 ± 4 pg/ml) (*p* < 0.05) (Figure 3C). These results indicated that empagliflozin treatment decreased myocardial injury after CA in rats.

### Empagliflozin Decreased Oxidative Stress After CA

**FIGURE 4:**
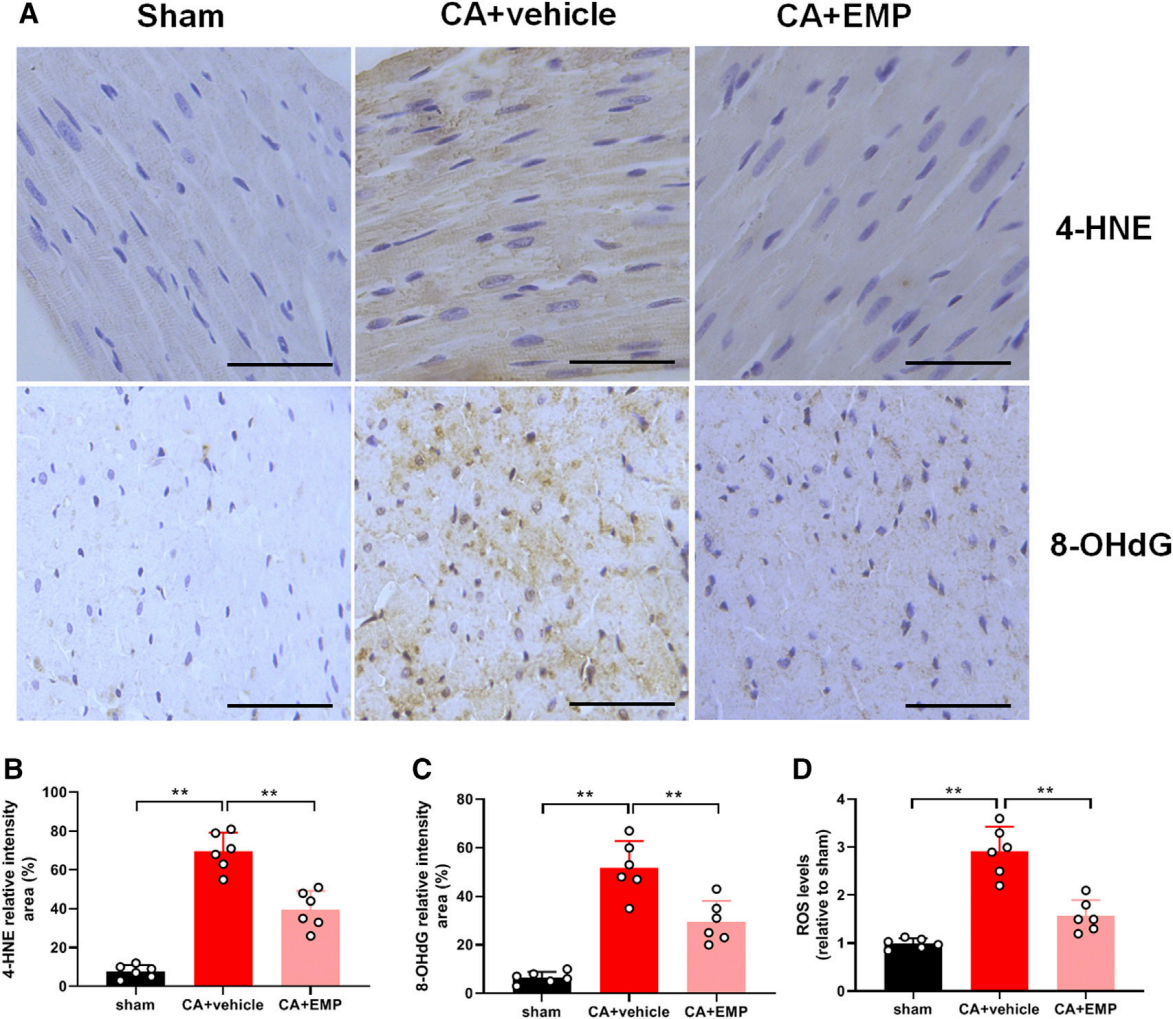
Empagliflozin (EMP) decreased myocardial oxidative stress after cardiac arrest in rats. **(A)** Representative photographs of 4-hydroxynonenal (4-HNE) and 8-hydroxy-2′-deoxyguanosine (8-OHdG) immunostaining of heart sections obtained 24 h after return of spontaneous circulation (ROSC). Scale bar = 50 μm. **(B)** Quantification of 4-HNE staining levels from stained heart sections. **(C)** Quantification of 8-OHdG staining levels from stained heart sections. **(D)** The relative reactive oxygen species (ROS) levels in heart tissues obtained 24 h ROSC. Data are presented as mean ± SD, *n* = 6, *p < 0.05, **p < 0.01.

Immunohistochemistry for 4-HNE (the end product of lipid peroxidation) and 8-OHdG (an index of oxidative DNA damage) was carried out to investigate oxidative stress in myocardium at 24 h after CA. The 4-HNE positive cardiomyocytes and 8-OHdG positive cells were distributed throughout the myocardium after CA (Figure 4A). Notably, there were fewer 4-HNE positive cardiomyocytes and 8-OHdG positive cells obtained from the rats administered empagliflozin (4-HNE: 40 ± 10% versus 70 ± 10%, *p* < 0.01; 8-OHdG: 30 ± 9% versus 52 ± 11%, *p* < 0.01) (Figures 4B,C). Moreover, the myocardial ROS levels were detected in three groups at 24 h after CA. The myocardial ROS levels increased significantly after ROSC (*p* < 0.01). However, the myocardial ROS levels in the CA + EMP group were significantly decreased compared with the CA + vehicle group (1.6 ± 0.3 versus 2.9 ± 0.5, *p* < 0.01) (Figure 4D). These results indicated that empagliflozin treatment ameliorated oxidative myocardial injury after CA in rats.

### Empagliflozin Protected Mitochondrial Ultrastructural Integrity After CA

**FIGURE 5:**
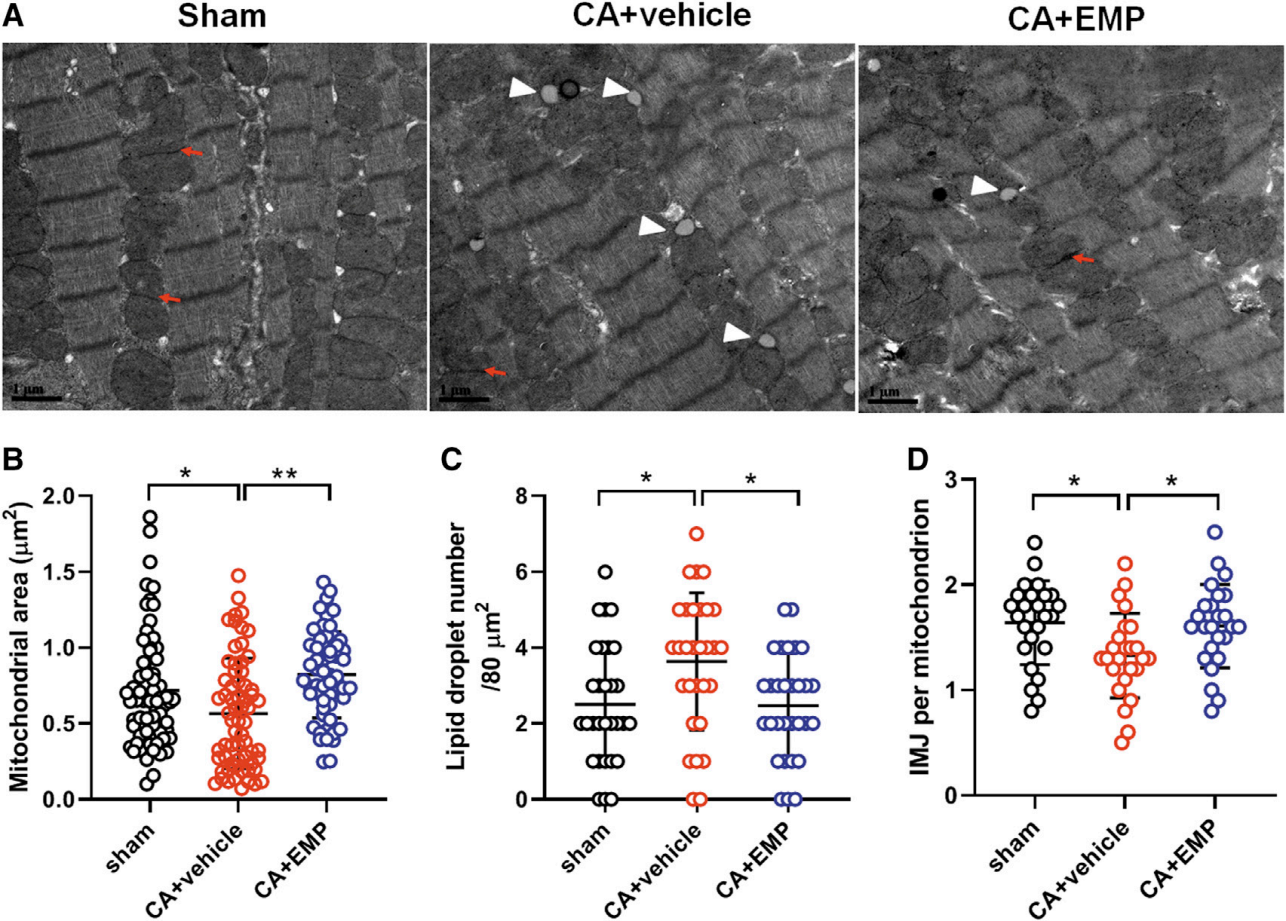
Effect of empagliflozin (EMP) on structural integrity of the myocardial mitochondria after cardiac arrest in rats. **(A)** Representative heart transmission electron microscopy (TEM) images of heart mitochondria at 24 h after return of spontaneous circulation (ROSC). Arrow indicates intermitochondrial junctions (IMJs); Arrowhead indicates lipid droplet. Scale bar = 1 μm. **(B)** Quantification of heart mitochondrial area by TEM after ROSC. *n* = 5 and at least 60 mitochondria were counted in each group. **(C)** Quantification of heart mitochondrial lipid droplet content by TEM after ROSC. *n* = 5 and 30 photographs were counted in each group. **(D)** Quantification of heart IMJs by TEM after ROSC. *n* = 5 and 25 photographs were counted in each group. Data are presented as mean ± SD, *p < 0.05, **p < 0.01.

The structure and integrity of mitochondria are essential for the physiological function of the heart. To determine the effect of empagliflozin on mitochondrial ultrastructure after CA, the myocardial tissue was examined at 24 h after CA by transmission electron microscopy. The electron microscopy analysis showed that mitochondria appeared smaller in the CA + vehicle group compared with mitochondria from the sham group, suggesting mitochondrial fission after CA (Figure 5A). However, empagliflozin treatment significantly decreased mitochondrial area after CA (mitochondrial area: 0.8 ± 0.3 μm^2^ versus 0.6 ± 0.4 μm^2^, *p* < 0.01) (Figure 5B). In addition, empagliflozin significantly reduced the protein expression of Drp1, a protein that regulates mitochondrial fission (*p* < 0.05) (Supplementary Figure S1). Moreover, the total number of lipid droplets in cardiomyocytes was significantly increased after CA. However, empagliflozin treatment significantly decreased the total number of lipid droplets after CA (2.5 ± 1.4 versus 3.6 ± 1.8, *p* < 0.05) (Figure 5C). The number of intermitochondrial junctions (IMJs) is an index of highly active mitochondria (Picard et al., 2015). Our results found that the number of IMJs in cardiomyocytes was significantly decreased after CA, but empagliflozin treatment significantly increased the number of IMJs after CA (1.6 ± 0.4 versus 1.3 ± 0.4, *p* < 0.05) (Figure 5D). These results demonstrated that empagliflozin treatment maintained the structural integrity of myocardial mitochondria after CA.

### Empagliflozin Increased Myocardial Energy Metabolism and Mitochondrial Activity After CA

**FIGURE 6:**
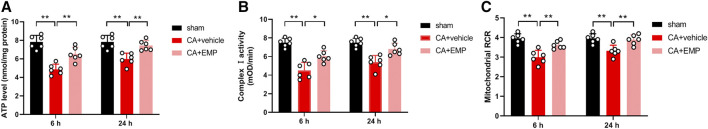
Empagliflozin (EMP) increased myocardial energy levels and mitochondrial activity after cardiac arrest in rats. **(A)** EMP increased myocardial ATP levels at 6 and 24 h after return of spontaneous circulation (ROSC). **(B)** EMP increased the myocardial mitochondrial complex Ⅰ activity at 6 and 24 h after ROSC. **(C)** EMP increased the myocardial mitochondrial respiratory control ratio (RCR) at 6 and 24 h after ROSC. Data are presented as mean ± SD, *n* = 6, *p < 0.05, **p < 0.01.

Alterations in mitochondrial bioenergetics play a key role in heart failure (Fillmore and Lopaschuk, 2013). Our results found that the myocardial ATP levels were significantly decreased at 6 and 24 h after ROSC, but empagliflozin treatment significantly increased the myocardial ATP levels at 6 and 24 h after ROSC (6 h: 6.5 ± 0.7 nmol/mg protein versus 4.9 ± 0.6 nmol/mg protein, *p* < 0.01; 24 h: 7.4 ± 0.5 nmol/mg protein versus 5.9 ± 0.6 nmol/mg protein, *p* < 0.01) (Figure 6A). We next investigated whether empagliflozin could affect mitochondrial activity after resuscitation. We found that empagliflozin treatment significantly increased the myocardial mitochondrial complexⅠactivity at 6 and 24 h after ROSC, respectively (6 h: 6.0 ± 0.6 mOD/min versus 4.5 ± 0.9 mOD/min, *p* < 0.05; 24 h: 6.8 ± 0.6 mOD/min versus 5.4 ± 0.7 mOD/min, *p* < 0.05) (Figure 6B). In addition, empagliflozin treatment significantly increased the myocardial mitochondrial RCR at 6 and 24 h after ROSC, respectively (6 h: 3.6 ± 0.2 versus 3.0 ± 0.3, *p* < 0.05; 24 h: 3.9 ± 0.3 versus 3.3 ± 0.3, *p* < 0.01) (Figure 6C). Therefore, these results suggested that empagliflozin treatment increased myocardial energy production and mitochondrial activity.

### Empagliflozin Increased Myocardial Ketone Metabolism After CA

**FIGURE 7:**
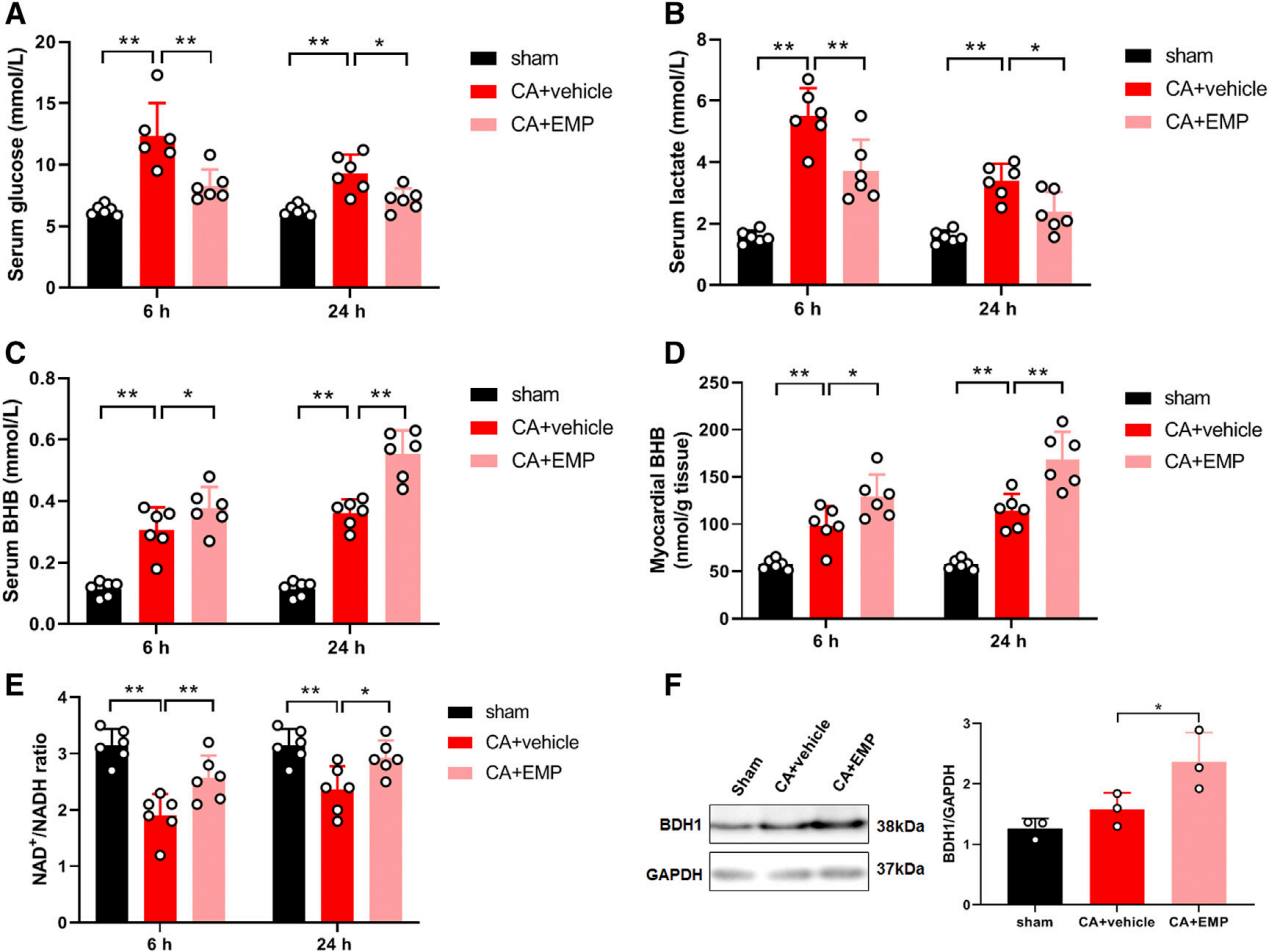
Empagliflozin (EMP) increased myocardial ketone metabolism after cardiac arrest in rats. **(A)** EMP decreased serum glucose levels at 6 and 24 h after return of spontaneous circulation (ROSC). **(B)** EMP decreased serum lactate levels at 6 and 24 h after ROSC. **(C)** EMP increased the serum β-hydroxybutyrate (BHB) levels at 6 and 24 h after ROSC. **(D)** EMP increased the myocardial BHB levels at 6 and 24 h after ROSC. **(E)** EMP increased the myocardial NAD^+^/NADH levels at 6 and 24 h after ROSC. **(F)** EMP increased the heart β-hydroxybutyrate dehydrogenase (BDH1) protein expression at 24 h after ROSC. GAPDH was used as the loading control. Data are presented as mean ± SD, *n* = 3 or 6, *p < 0.05, **p < 0.01.

We investigated whether empagliflozin could affect glucose metabolism after resuscitation. The results showed that serum glucose levels were significantly increased at 6 and 24 h after ROSC, respectively. However, empagliflozin treatment significantly decreased serum glucose levels at 6 and 24 h (6 h: 8.3 ± 1.3 mmol/L versus 12.4 ± 2.7 mmol/L, *p* < 0.01; 24 h: 7.2 ± 0.9 mmol/L versus 9.3 ± 1.5 mmol/L, *p* < 0.05) (Figure 7A). In addition, empagliflozin treatment significantly decreased serum lactate levels at 6 and 24 h (6 h: 3.7 ± 1.0 mmol/L versus 5.5 ± 0.9 mmol/L, *p* < 0.01; 24 h: 2.4 ± 0.7 mmol/L versus 3.4 ± 0.6 mmol/L, *p* < 0.05) (Figure 7B). Ketones are an important alternative fuel for the cell under glucose hypometabolic conditions. To determine the role of ketones in empagliflozin mediated energy metabolism, BHB concentrations were detected in the serum and heart at 6 and 24 h after CA. The serum BHB concentrations were significantly increased after empagliflozin treatment at 6 and 24 h after ROSC, respectively (6 h: 0.38 ± 0.07 mmol/L versus 0.31 ± 0.07 mmol/L, *p* < 0.05; 24 h: 0.55 ± 0.08 mmol/L versus 0.36 ± 0.04 mmol/L, *p* < 0.01) (Figure 7C). Similarly, empagliflozin treatment significantly increased the myocardial BHB concentrations at 6 and 24 h after ROSC, respectively (6 h: 129 ± 23 nmol/g tissue versus 99 ± 21 nmol/g tissue, *p* < 0.05; 24 h: 169 ± 29 nmol/g tissue versus 114 ± 18 nmol/g tissue, *p* < 0.01) (Figure 7D). Ketone-based metabolism increases availability of NAD^+^ and thus alters the NAD^+^/NADH ratio (Elamin et al., 2017). Our data showed that the myocardial NAD^+^/NADH ratios were significantly increased after empagliflozin treatment at 6 and 24 h after ROSC, respectively (6 h: 2.6 ± 0.4 versus 1.9 ± 0.4, *p* < 0.01; 24 h: 2.8 ± 0.5 versus 2.4 ± 0.4, *p* < 0.05) (Figure 7E). To determine whether the increases in ketone levels were associated with changes in the myocardial capacity to utilize ketone bodies, a critical protein named BDH1 involved in myocardial ketolysis was detected. The protein expression of BDH1 was significantly increased after empagliflozin treatment at 24 h after ROSC (*p* < 0.05) (Figure 7F). Taken together, these results demonstrated that empagliflozin treatment increased the myocardial ketone metabolism after CA.

## Discussion

Empagliflozin is a newly approved antidiabetic drug that inhibits SGLT2 in an insulin-independent manner. In addition to its excellent glucose-lowering effect, recent studies demonstrated that empagliflozin markedly improved myocardial function in diabetic and non-diabetic heart failure (Fitchett et al., 2016; Santos-Gallego et al., 2019; Yurista et al., 2019; Packer et al., 2020). However, whether empagliflozin improves cardiac outcomes in myocardial injury after CA is unknown. In the present study, we investigated the effects of empagliflozin on myocardial injury in rats using a VF induced CA and CPR model. Maintaining hemodynamics and preventing circulatory failure are important for early postresuscitation care (Chang et al., 2007). Although empagliflozin did not improve the heart rate and blood pressure after resuscitation, it did not worsen the heart hemodynamics within 1 h after resuscitation in our results. We found that empagliflozin treatment significantly increased the cardiac LVEF, CO and survival time of the animals at 24 h after CA. In addition, empagliflozin decreased myocardial fibrosis and serum cTnI levels after resuscitation. Therefore, our findings suggested that empagliflozin had a protective effect on myocardial injury after CA in non-diabetic rats.

It has long been recognized that reoxygenation of myocardial tissue results in an increase in the production of oxidative stress due to excess ROS (Patil et al., 2015; Yang et al., 2015). The mitochondria are major sources of ROS within the cell and the uncontrolled of mitochondria upon reoxygenation with subsequent ROS generation has deleterious effects on numerous macromolecules, such as proteins, lipids and DNA, leading to impaired cell function. ROS can lead to DNA damage and formation of 8-OHdG, a prominent feature in diabetic hearts (Nishio et al., 2012). ROS can directly damage mitochondrial macromolecules and mitochondrial DNA, which will affect the normal functions of mitochondria, such as energy metabolism. Lipid oxidation by ROS results in the formation of carbonyl compounds, such as 4-HNE, toxic metabolites that can promote numerous pathologies. The detrimental ROS mediates myocardial injury and cardiomyocyte death through a number of different mechanisms. Damaged mitochondria result in enhanced ROS production and activation of the NLRP3 inflammasome, which may promote or exacerbate cardiac fibrosis (Zhou et al., 2011). Moreover, ROS can modulate sarcomere function by affecting key proteins forming the thick and thin filaments (Santos et al., 2011). Our study showed that 4-HNE and 8-OHdG positive cardiomyocytes were significantly increased after ROSC, indicating high oxidative stress in the heart. However, empagliflozin treatment ameliorated oxidative myocardial injury after CA. Serval studies have demonstrated that hyperglycemia-induced pathogenic mechanisms contributed to ROS production, such as glucose auto-oxidation, metabolism, and formation of advanced glycosylation end products (Kaludercic and Di Lisa, 2020; Lu et al., 2020). Given that hyperglycemia occurs after CA, the effect of empagliflozin on myocardial oxidative stress after CA may be associated with reduction of glucose level. Furthermore, it has reported that empagliflozin suppressed myocardial oxidative stress through activation of Nrf2/ARE signaling in diabetic mice (Li et al., 2019).

Mitochondria are remarkable organelles capable of remodeling their structural organization to optimize mitochondrial function (Glancy et al., 2020). In our study, we found that the structure and integrity of mitochondria in heart were changed after CA such as mitochondrial fission and the number of lipid droplets, and empagliflozin treatment maintained the structural integrity of myocardial mitochondria after CA. Mitochondrial quality control mechanisms are largely regulated by mitochondrial dynamics, including fission (division) and fusion. Mitochondrial fission creates smaller, more discrete mitochondria, which are more capable of generating ROS and leads to cell apoptosis. Previous studies have demonstrated that inhibition of mitochondrial fission protected the heart against ischemia/reperfusion injury (Ong et al., 2010; Sharp et al., 2015). Lipid droplets are cytosolic organelles composed of fatty acids. Mitochondria-lipid droplet interactions represent a significant mitochondrial network structure, and these interactions are dynamic. The function of mitochondria-lipid interactions may support either metabolism or synthesis of fatty acids depending on the cell type and physiology of the cells (Glancy et al., 2020). Overabundant and/or enlarged lipid droplets are the hallmarks of obesity, type 2 diabetes, cardiac steatosis, and cardiomyopathy (Krahmer et al., 2013). It has reported that accumulation of lipids in myocardial lipid droplets was associated with heart failure in obesity and diabetes mellitus (Sharma et al., 2004). Hyperglycemia may play an important role in mitochondrial dynamic and lipid droplets accumulation. It has reported that hyperglycemia enhances the ischemia-induced mitochondrial fission by ROCK1 activation and Drp1 translocation to the mitochondria (Wang et al., 2012). Furthermore, study showed that high glucose concentration increases the amount of lipid droplets on colorectal cells (Tirinato et al., 2020).

Alterations in mitochondrial energy metabolism are common in many forms of heart disease, including heart failure, ischemic heart disease and diabetic cardiomyopathies (Fillmore et al., 2014). In healthy heart, 60–90% of ATP production comes from β-oxidation of fatty acids, whereas 10–40% comes from oxidation of pyruvate derived from glycolysis (Yang et al., 2015). Fatty acids are the major source of myocardial energy metabolism but have high oxygen requirements. During myocardial ischemia, glucose becomes the preferred myocardial energy metabolism substrate because it is more oxygen efficient than fatty acids, but this is at the expense of lower energy produced (Fillmore and Lopaschuk, 2013; Fillmore et al., 2014). At that time, pyruvate produced by glycolysis is not oxidized via oxidative phosphorylation in mitochondria, but rather is reduced to lactate in the cytosol. The increase in glycolytic rate results in anaerobic production of ATP and excess cytosolic protons, leading to intracellular acidosis. The increase in lactate and proton production has the potential to be detrimental to the heart. The serum lactate level is a good prognostic predictor for CA patients and elevation of lactate concentration has been associated with increased mortality after out-of-hospital CA (Shinozaki et al., 2011). Therefore, the shift from in myocardial energy metabolism from fatty acids to glucose consumption leads to an energy deficit and lactate accumulation that impairs cardiac efficiency. Thus, a reduction of ATP level, decrease of mitochondrial complexⅠactivity, and accumulation of lipid droplets and lactate were observed in heart after CA in our study. Ketone bodies are an alternative fuel when energy is insufficient, and are more efficient than fatty acids on the basis of ATP produced per oxygen consumed (P/O ratio) (Lopaschuk et al., 2020). Moreover, ketone bodies as a substrate for mitochondrial energy metabolism have no lactate production. Our study found that empagliflozin treatment significantly increased myocardial ATP levels, ketone bodies and mitochondrial activity after CA. In addition, ketone body such as BHB can reduce production of ROS via action at complex II (Achanta and Rae, 2017). Recent studies have demonstrated that ketone body metabolism significantly protected the failing heart (Lopaschuk et al., 2020; Schulze and Wu, 2020). Therefore, our results suggested that the protective effects of empagliflozin on myocardial energetics and cardiac function after CA were mediated by ketone body metabolism.

We acknowledged that our study had some limitations. First, we did not include an empagliflozin-treated sham group because our primary focus was the effects of empagliflozin on myocardial dysfunction after CA. Second, our study focused on the left ventricular systolic function after CA, but the diastolic characteristics of post-CA myocardial dysfunction were not defined. In future studies, the effect of empagliflozin on diastolic function of the heart after CA would be examined. Third, our study mainly studied the early myocardial injury after CA (6 and 24 h of CA). Further studies should prolong the observation period to assess the effect of empagliflozin. Finally, we used only adult male rats for animal model in our study, but CA also occurs in both elderly males and females clinically. Future studies should be investigated in aged and female rats to better simulate the human condition.

In conclusion, our data demonstrated that SGLT2 inhibition with empagliflozin ameliorated myocardial fibrosis, enhanced left ventricular function, and increased survival time in a non-diabetic rat model ventricular fibrillation CA and CPR. These cardiac benefits of empagliflozin seem to be mediated by reducing glucose levels and increasing ketone body oxidized metabolism, which improves myocardial energetics and cardiac function. Our findings highlight the therapeutic potential of empagliflozin for myocardial dysfunction after CA and warrant further investigation.

## Data Availability

The original contributions presented in the study are included in the article/Supplementary Material, further inquiries can be directed to the corresponding authors.

## Supplementary Material

Supplementary Figure S1 Empagliflozin (EMP) decreased myocardial Drp1 protein expression after cardiac arrest in rats. **(A)** EMP decreased the heart Drp1 expression at 24 h after ROSC. GAPDH was used as the loading control. **(B)** Quantification of heart Drp1 protein expression by western blot band intensity measurement. Data are presented as mean ± SD, *n* = 3, *P < 0.05.
