# Supplementary material for: Sodium–Glucose Co-Transporter 2 Inhibition With Empagliflozin Improves Cardiac Function After Cardiac Arrest in Rats by Enhancing Mitochondrial Energy Metabolism
Source: Front Pharmacol. 2021 Oct 12;12:758080. doi: 10.3389/fphar.2021.758080 (PMC8546214; doi:10.3389/fphar.2021.758080)
Supplement: Supplementary file 1 [file Table1.DOCX]

| **Variables** | **Sham**  **(n = 6)** | **CA+vehicle**  **(n = 22)** | **CA+EMP**  **(n = 18)** |
| --- | --- | --- | --- |
| Weight(g） | 340 ± 10 | 342 ± 12 | 344 ± 11 |
| Heart rate (beats/min) | 386 ± 21 | 379 ± 25 | 383 ± 27 |
| MAP (mm Hg) | 132 ± 6 | 136 ± 4 | 133 ± 6 |
| Rectal temperature (°C) | 36.4 ± 0.3 | 36.7 ± 0.4 | 36.5 ± 0.3 |
| ETCO_2_ (mm Hg) | 35.6 ± 3.2 | 36.2 ± 3.5 | 35 ± 3.8 |
| Arterial lactate (mmol/L) | 1.1 ± 0.13 | 1.05 ± 0.1 | 1.08 ± 0.16 |
| Arterial pH | 7.42 ± 0.03 | 7.41 ± 0.04 | 7.43 ± 0.04 |
| EF (%) | 71 ± 8 | 68 ± 7 | 70 ± 7 |
| FS (%) | 45 ± 7 | 43 ± 6 | 41 ± 6 |
| Duration of PC (s) |  | 251 ± 31 | 254 ± 35 |
| CPP in PC1 |  | 16.5 ± 2.5 | 16.2 ± 3.1 |
| CPP in PC3 |  | 30.2 ± 8.9 | 30.8 ± 8.4 |

**Supplementary Table 1 Baseline and CPR characteristics of three groups**

Values are presented as mean ± SD. *CPR* cardiopulmonary resuscitation group, *CA* cardiac arrest, *EMP* empagliflozin, *MAP* mean artery pressure, *ETCO2* end-tidal CO_2_, *EF* ejection fraction, *FS* fractional shortening, *PC* precordial compression, *CPP* coronary perfusion pressure, *PCn*, n minutes after precordial compression.
