# Supplementary figures and images for: Sodium–Glucose Co-Transporter 2 Inhibition With Empagliflozin Improves Cardiac Function After Cardiac Arrest in Rats by Enhancing Mitochondrial Energy Metabolism
Source: Front Pharmacol. 2021 Oct 12;12:758080. doi: 10.3389/fphar.2021.758080 (PMC8546214; doi:10.3389/fphar.2021.758080)

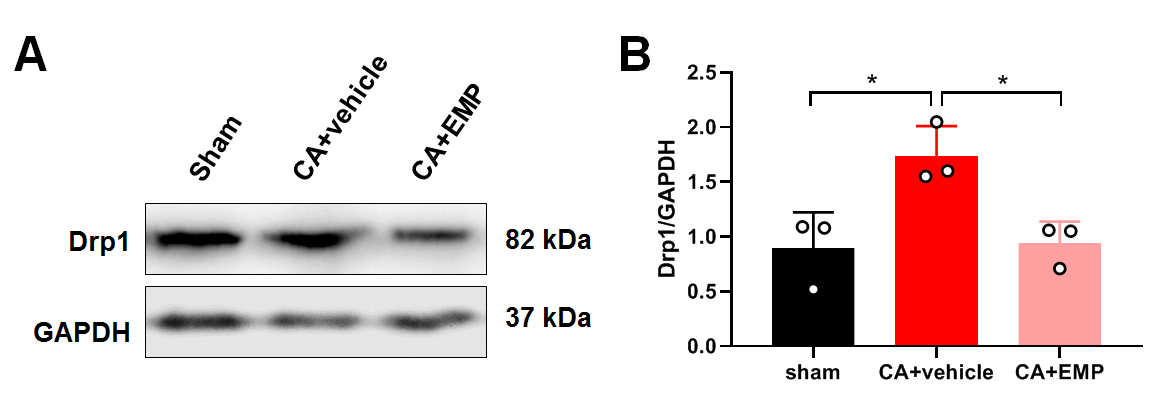

Supplement: Supplementary file 2 [file Image1.TIF]
